# Effects of Supplemental Lighting on Potassium Transport and Fruit Coloring of Tomatoes Grown in Hydroponics

**DOI:** 10.3390/ijms22052687

**Published:** 2021-03-07

**Authors:** Wei Wang, Danxia Liu, Min Qin, Zhenbin Xie, Riyuan Chen, Yiting Zhang

**Affiliations:** College of Horticulture, South China Agricultural University, Guangzhou 510642, China; weiwang@stu.scau.edu.cn (W.W.); liudanxia@stu.scau.edu.cn (D.L.); qinmin@stu.scau.edu.cn (M.Q.); xj-99gzxwz@stu.scau.edu.cn (Z.X.)

**Keywords:** supplemental lighting, potassium, fruit coloring, potassium transporters

## Abstract

Supplemental blue/red lighting accelerated fruit coloring and promoted lycopene synthesis in tomato fruits. Potassium (K) is the most enriched cation in tomato fruits, and its fertigation improved tomato yield and fruit color. However, the effects of supplemental lighting on K uptake and transport by tomatoes and whether supplemental lighting accelerates fruit coloring through enhancing K uptake and transport are still unclear. We investigated the effects of supplemental light-emitting diode (LED) lighting (SL; 100% red, 100% blue; 75% red combined 25% blue) on K uptake in roots and transport in the fruits as well as the fruit coloring of tomatoes (Micro-Tom) grown in an experimental greenhouse in hydroponics. The use of red SL or red combined blue SL enhanced K uptake and K accumulation as well as carotenoid (phytoene, lycopene, γ-carotene, and β-carotene) content in fruits by increasing photosynthesis, plant growth, and fruit weight. The genes related to ethylene signaling were upregulated by red SL. Quantitative real-time PCR (qRT-PCR) results showed that K transporter genes (*SlHAK*s) are differentially expressed during fruit development and ripening. The highest-expressed gene was *SlHAK10* when fruit reached breaker and ripening. *SlHAK3* and *SlHAK19* were highly expressed at breaker, and *SlHAK18* was highly expressed at ripening. These might be related to the formation of tomato fruit ripening and quality. *SlHAK4*, *SlHAK6, SlHAK8,* and *SlHAK9* were significantly downregulated with fruit ripening and induced by low K. The expression level of *SlHAK6, SlHAK10*, *SlHAK15,* and *SlHAK19* were significantly increased by blue SL or red combined blue SL during breaker and ripening. Blue SL or red combined blue SL increased content of phytoene, β-carotene, α-carotene, and γ-carotene and accelerated fruit coloring by enhancing K uptake in roots and transport in fruits during fruit ripening. This was consistent with the expression level of *SlHAK6*, *SlHAK10*, *SlHAK15*, and *SlHAK19* during fruit development and ripening. The key genes of photoreceptors, light signaling transcript factors as well as abscisic acid (ABA) transduction induced by blue SL or red combined blue SL were consistent with the upregulated genes of *SlHAK6*, *SlHAK10*, *SlHAK15,* and *SlHAK19* under blue SL and red combined blue SL. The K transport in tomato fruits might be mediated by light signaling and ABA signaling transduction. These results provide valuable information for fruit quality control and the light regulating mechanism of K transport and fruit coloring in tomatoes.

## 1. Introduction

Tomato (*Solanum lycopersicum* L.) is one of the most important horticultural crops in China and year-round supply is desired by consumers. Fruit color is a key determinant of tomato fruit quality that largely affects the initial quality assessment by the consumer [1]. Fruit coloring disorder (slowly progressing or nonuniform coloring) often appears in greenhouse tomato after they suffered from continuous low solar irradiance or/and low temperature conditions, especially during winter to early-spring growing seasons in China, which seriously restricts the sustainable production of high quality tomatoes. 

In recent years, supplemental LED lighting (SL) has been used as an efficient light source to meet the demand for high irradiance during plant growth and fruit development and to maintain the productivity of greenhouse tomatoes, particularly during the low irradiance seasons [2,3,4,5,6]. We accelerated the tomato fruit coloring process, and promoted lycopene synthesis in tomato fruits by applying supplemental blue or/and red light during winter to early-spring growing seasons [7]. SL increased lycopene content in tomatoes by inducing light receptors that modulate HY5 and PIFs activation to mediate the expression of key genes in lycopene synthesis [8]. SL results in the earlier ripening of tomato fruit depending on ethylene production [9]. 

Potassium (K) is the most enriched cation in tomato [10], and its requirement is extraordinarily high due to the rapid growth of the plant in combination with the heavy fruit load [11,12]. Tomato fruits exhibiting coloring disorder often contain lower K concentration than normal fruit [13], but increasing K fertilizer can effectively improve tomato fruit coloring disorder, which is caused by poor temperature and light environment [14,15]. The lycopene content in tomato fruit increased linearly with increasing K level in the nutrient solution [16]. In our previous studies, the occurrence of coloring disorder in tomato fruit was significantly related with K uptake. Particularly during high fruit-load stage, K uptake is the key to tomato fruit coloring [15]. 

Light was thought to modulate the uptake and utilization of mineral elementals such as nitrogen [17], phosphorus [18], sulfur [19], and copper [20] by inducing light receptors that modulate transcription factors activation to mediate expression of transporter genes in *Arabidopsis thaliana*. However, the regulatory mechanism of light on the absorption and utilization of K has not been reported. Several studies have explored the functional and regulation of K transporters in fruit ripening and coloring such as grape berry [21], peach [22,23], and strawberry fruit [24]. However, little research is available on K transporters in tomato, especially in the fruits. Nineteen KT/HAK/KUP family genes were identified in tomatoes [25]. 

To understand the role of supplemental red and blue (LED) light on K uptake and transport as well as fruit coloring of tomatoes, ‘Micro-Tom’ tomatoes were treated with supplementary red (660 nm), blue (430 nm), and red and blue (3:1) LED lighting under two KNO_3_ supply levels (405 mg/L and 101mg/L) in hydroponic cultivation, respectively. We analyzed K uptake rate, fruit K content, fruit coloring, carotenoid content, and photosynthesis as well as the chlorophyll fluorescence parameters. Moreover, gene expression levels of K transporter, light receptors, and light signaling components were investigated and transcriptome analysis was carried out to provide the molecular mechanism. 

## 2. Results

### 2.1. Characteristics of Plant Growth and K Content in Different Tissues

**Figure 1:**
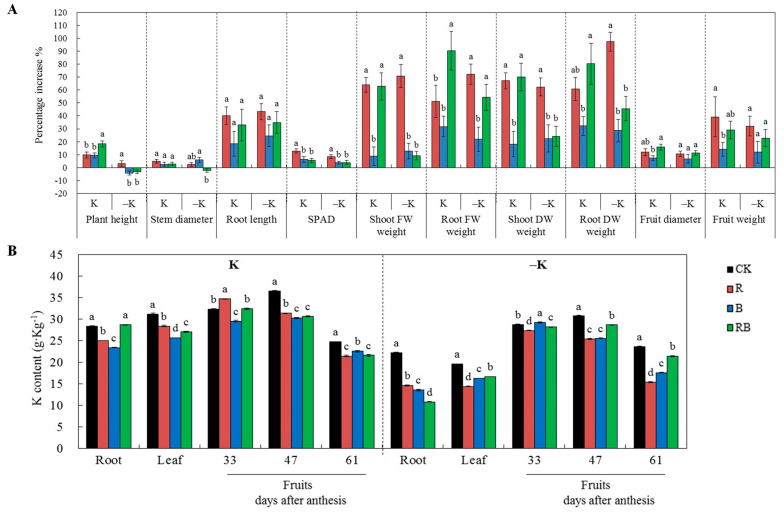
Plant growth characteristics and K content of tomatoes grown under red or/and blue supplemental lighting or without supplemental lighting at low K or normal K level. (**A**) The increase percentage of growth. (**B**) K content in tomato root, leaf, and fruit at 33, 47, and 61 DAA (days after anthesis). Error bars represent standard deviations of the means of three independent replicates. Different letters indicate significant differences between treatments by Duncan’s multiple range test (*p* ≤ 0.05). FW: fresh weight, DW: dry weight, SPAD: soil plant analysis development, leaf chlorophyll content, CK: natural light, R: supplemental 660 nm red light, B: supplemental 430 nm blue light, RB: supplemental red combined with blue light with ratio of 3:1, K: normal potassium supply, –K: low potassium supply.

As shown in Figure 1A, except for plant height, which was reduced by blue supplemental lighting (SL), the other growth characteristics were enhanced with SL (Figure 1A). The percentage increase was higher in red or red combined SL compared with blue SL at normal or low K supply, suggesting that red light was more effective than blue light for plant growth. Fruit accumulated more K than root and leaves in tomato (Figure 1B). K content in tomato fruits was the highest at 47 DAA at normal K supply, but decreased with ripening under SL treatments (Figure 1B). K content in roots, leaves, and fruits decreased with SL at both K levels (Figure 1B). Red SL significantly increased K content in tomato fruit at 33 DAA compared with CK at normal K supply, whereas blue SL increased K content in tomato fruit at 33 DAA at low K supply (Figure 1B) compared with CK. The K content in tomato fruits at 61 DAA was higher in blue SL than in red SL treatment (Figure 1B). 

### 2.2. Characteristics of Photosynthesis, Maximum Quantum Efficiency of Photosystem Ⅱ, and Non-photochemical Quenching.

**Figure 2:**
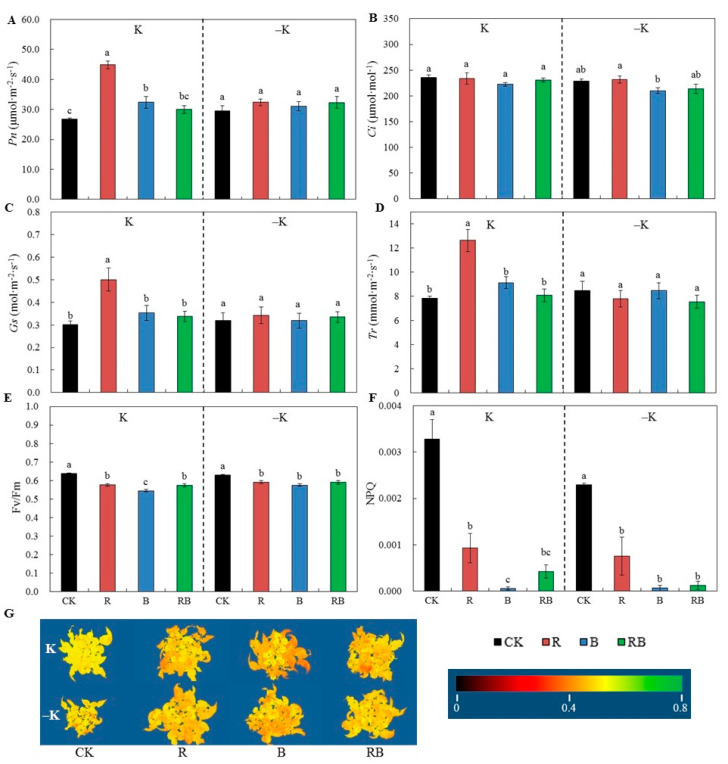
Photosynthesis characteristics and photochemistry efficiency of photosystem Ⅱ (PSII) of tomatoes grown under red or/and blue supplemental lighting or without supplemental lighting at low K or normal K level. (**A**) Net photosynthetic rates (*Pn*); (**B**) Stomatal conductance (*Gs*); (**C**) Intercellular CO_2_ concentration (*Ci*); (**D**) Transpiration rate (*Tr*); (**E**) Maximum quantum efficiency of PSII (Fv/Fm); (**F**) Non-photochemical quenching (NPQ); (**G**) Whole plant images created using PlantExplorer^TM^ indicate Fv/Fm. Error bars represent standard deviations of the means of three independent replicates. Different letters indicate significant differences between treatments by Duncan’s multiple range test (*p* ≤ 0.05). CK: natural light, R: supplemental 660 nm red light, B: supplemental 430 nm blue light, RB: supplemental red combined with blue light with ratio of 3:1, K: normal potassium supply, –K: low potassium supply.

In comparison with CK, *Pn* was significantly increased with red SL or blue SL at normal K supply, and red SL was significantly higher than blue SL, which showed no significant difference at low K supply (Figure 2A). *Ci* was not affected by all SL (Figure 2B). The red SL increased *Gs* and *Tr* at normal K supply (Figure 2C,D). The maximum photochemical quantum yield of photosystem Ⅱ (PSII) (Fv/Fm) was reduced with SL at both K supply (Figure 2E), and the reduction in Fv/Fm was also visible from the acquired plant images (Figure 2G). The plant non-photochemical quenching (NPQ) was significantly reduced with SL, and the blue SL or red combined blue was lower than red SL (Figure 2F).

### 2.3. K Uptake Rate and Fruit Coloring

**Figure 3:**
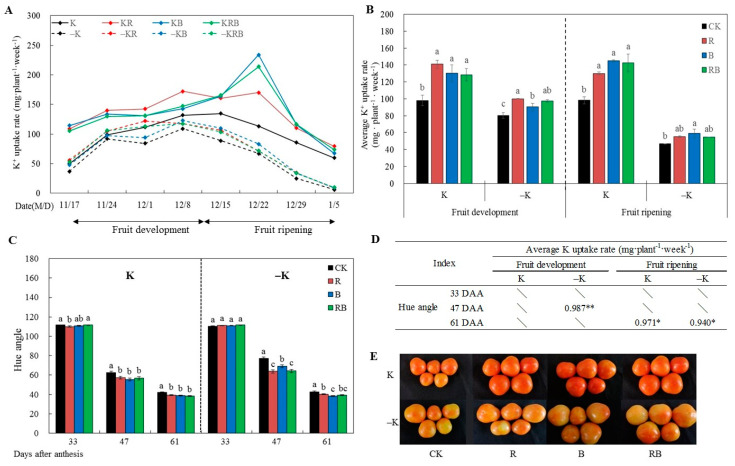
K uptake and fruit coloring of tomatoes grown under red or/and blue supplemental lighting or without supplemental lighting at low K or normal K level. (**A**) K uptake rate; (**B**) average K uptake rate during fruit development and ripening stage; (**C**) hue angle values of tomato fruits at 33, 47, 61 DAA; (**D**) correlation between K uptake and hue angle; (**E**) fruit coloring at 47 DAA. Error bars represent standard deviations of the means of three independent replicates. Different letters indicate significant differences between treatments by Duncan’s multiple range test (*p* ≤ 0.05). The asterisk * and ** indicate a significant difference at *p* ≤ 0.05, *p* ≤ 0.01, respectively. CK: natural light, R: supplemental 660 nm red light, B: supplemental 430 nm blue light, RB: supplemental red combined with blue light with ratio of 3:1, K: normal potassium supply, –K: low potassium supply.

Weekly variation of K uptake rate and fruit coloring were monitored during fruit development and ripening stages. As shown in Figure 3, weekly K uptake rate tended to increase during fruit development, then decreased during fruit ripening, while a short period of increase during fruit ripening was detected under SL combined at normal K. According to the difference of K uptake between both stages, we calculated the average K uptake rate as shown in Figure 3B. It was significantly increased in all SL treatments, and there was no significant differences among SL treatments at normal K supply (Figure 3B). However, at low K supply, average uptake rate of K in red SL treatment was significantly higher than that in blue SL treatment during the fruit development stage, while only blue SL increased the average uptake rate of K during the fruit ripening stage compared with CK (Figure 3B). The SL treatments significantly reduced the hue angle value of tomato fruit at 47 DAA and 61 DAA (Figure 3C). There was no significant difference among SL treatments under normal K supply. However, in the condition of low K supply, the hue angle value in red SL or red combined blue SL was significantly lower than that in blue SL of fruit at 47 DAA, while blue SL was lower than red SL of fruit at 61 DAA. The hue angle value at 47 DAA was correlated with the average K uptake rate under low K during the fruit development stage, while during the fruit ripening stage, the hue angle value was significantly correlated with the K uptake rate at both K supply levels (Figure 3D). Tomato plants cultivated in low K nutrients solution bore fruits with yellow spot, which was alleviated by SL treatments (Figure 3E).

### 2.4. Fruit Carotenoid Content

**Figure 4:**
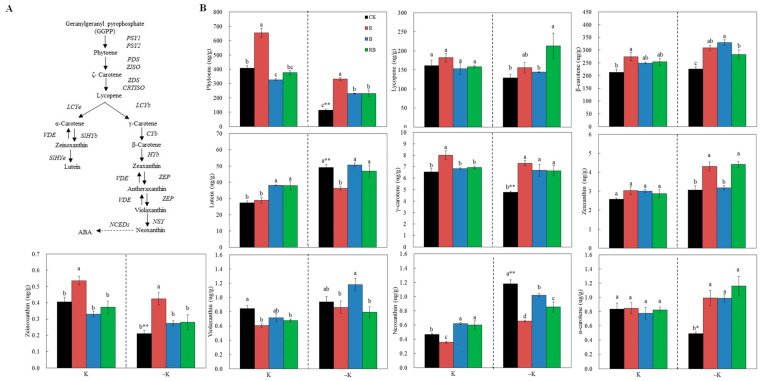
Carotenoid content analysis of tomatoes grown under red or/and blue supplemental lighting or without supplemental lighting at low K or normal K level. (**A**) The carotenoid biosynthetic pathway [26]; (**B**) carotenoid content in tomato fruit at 47 DAA (days after anthesis) grown under no supplemental lighting or red or/and supplemental lighting. Error bars represent standard deviations of the means of three independent replicates. Different letters indicate significant differences between treatments by Duncan’s multiple range test (*p* ≤ 0.05). The asterisk * and ** indicate a significant difference at *p* ≤ 0.05, *p* ≤ 0.01, respectively.CK: natural light, R: supplemental 660 nm red light, B: supplemental 430 nm blue light, RB: supplemental red combined with blue light with ratio of 3:1, K: normal potassium supply, –K: low potassium supply.

As differences of fruit coloring were detected at 47 DAA (Figure 3C), a quantitative analysis of carotenoids in the tomato fruits was carried out at 47 DAA (Figure 4). Under low K supply, the contents of phytoene, γ-carotene, α-carotene, and zeinoxanthin of tomato fruits were significantly decreased, while the contents of lutein and neoxanthin were significantly increased (Figure 4B). However, the content of phytoene, γ-carotene, α-carotene, and β-carotene content in tomato fruits was significantly increased by blue and red combined blue SL, and lycopene content significantly increased under red combined blue SL, whereas this phenomenon was not observed under normal K supply, indicating that SL of blue or red combined blue SL treatment probably enhanced carotenoid accumulation through enhancing K uptake under low K supply. The red SL increased the content of phytoene, γ-carotene, β-carotene, and zeinoxanthin, which was not related to the K supply level (Figure 4B). Neoxanthin content was reduced by red SL, and not related to the K supply level, whereas it was significantly increased by blue or red combined blue SL at normal K. Lutein content was increased by blue or red combined blue SL under normal K, whereas red SL significantly decreased lutein content under low K (Figure 4B).

### 2.5. Expression Analysis of Potassium Transporter and Channel Genes

**Figure 5:**
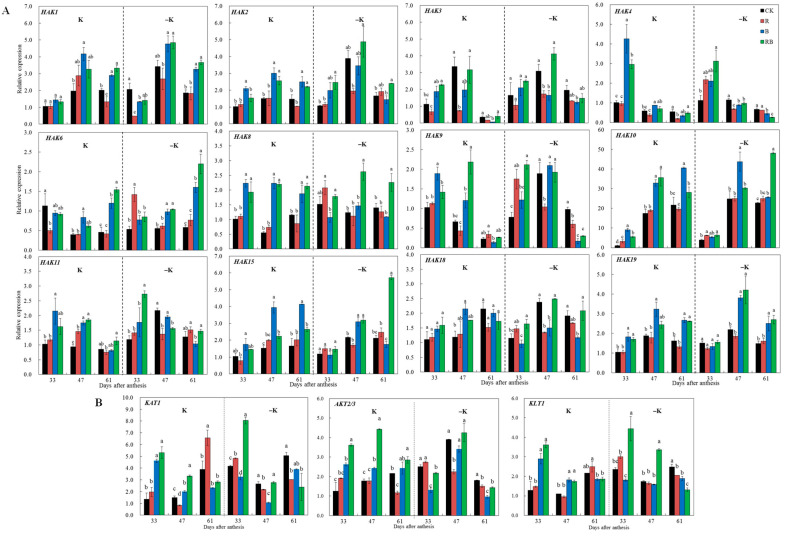
Transcriptional analysis of the potassium transporter genes *SlHAKs* and channel genes at 33, 47, and 61 DAA (days after anthesis) grown under red or/and blue supplemental lighting or without supplemental lighting at low K or normal K level. (**A**) *SlHAKs* expression patterns; (**B**) K channel expression patterns. The expression was normalized to the 33 DAA CK sample. Error bars represent standard deviations of the means of three independent replicates. Different letters indicate significant differences between treatments by Duncan’s multiple range test (*p* ≤ 0.05). CK: natural light, R: supplemental 660 nm red light, B: supplemental 430 nm blue light, RB: supplemental red combined with blue light with ratio of 3:1, K: normal potassium supply, –K: low potassium supply.

A quantitative real-time PCR (qRT-PCR) was used to analyze the transcriptional levels of K transporter KT/HAK/KUP (*SlHAKs*) family genes in tomatoes as well as the response to low K and supplemental lighting (Figure S2; Figure 5). The *SlHAK10* was highly expressed at 47 DAA and 61 DAA, which reached nearly 10-fold higher than the others when fruits reached breaker and ripening. The *SlHAK3* and *SlHAK19* were highly expressed at 47 DAA, and *SlHAK18* was highly expressed at 61 DAA (Figure S2). Thus, *SlHAK1*, *SlHAK3*, *SlHAK10*, and *SlHAK19* might belong to the same gene class in regulating K transport and fruit coloring of tomato fruits. In contrast, *SlHAK4*, *SlHAK6, SlHAK8,* and *SlHAK9* were significantly downregulated with fruit ripening. *SlHAK2, SlHAK4, SlHAK6, SlHAK8, SlHAK9, SlHAK11, SlHAK15*, and *SlHAK18* were significantly induced by low K supply at 47 DAA (Figure S2). 

Under both K levels, the expression levels of *SlHAK6, SlHAK10*, *SlHAK15*, and *SlHAK19* were significantly higher in blue SL when fruits reached the breaker stage at 47 DAA compared with no SL (Figure 5A). *SlHAK1*, *SlHAK6*, and *SlHAK19* were upregulated in blue SL or red combined blue SL treatment at 61 DAA. *SlHAK6* and *SlHAK19* were upregulated by blue SL from the fruit breaker to ripening stages, suggesting that these K transporter genes were regulated by blue or red combined blue SL, which was not related to the K level. The blue SL induced *SlHAK2*, *SlHAK3*, *SlHAK4*, *SlHAK8, SlHAK9*, *SlHAK11,* and *SlHAK19* at 33 DAA, but there was no significant difference under low K supply compared with CK. Similarly, under normal K supply, the expression levels of *SlHAK3*, *SlHAK4*, *SlHAK8*, and *SlHAK10* were significantly higher in red combined blue SL treatment at 33 DAA. The expression levels of *SlHAK2*, *SlHAK7*, *SlHAK8, SlHAK10*, *SlHAK11*, and *SlHAK15* were significantly higher at 47 DAA in blue and red combined blue SL treatments compared with CK (Figure 5A). *KAT1* was upregulated by red SL at 61 DAA compared with CK (Figure 5B). *AKT2/3* and *KLT1* were highly induced by red combined blue SL at the normal K supply level (Figure 5B). *KLT1* was also upregulated by blue at 33 and 47 DAA.

### 2.6. Expression Analysis of Genes Related to the Light Signaling Pathway

**Figure 6:**
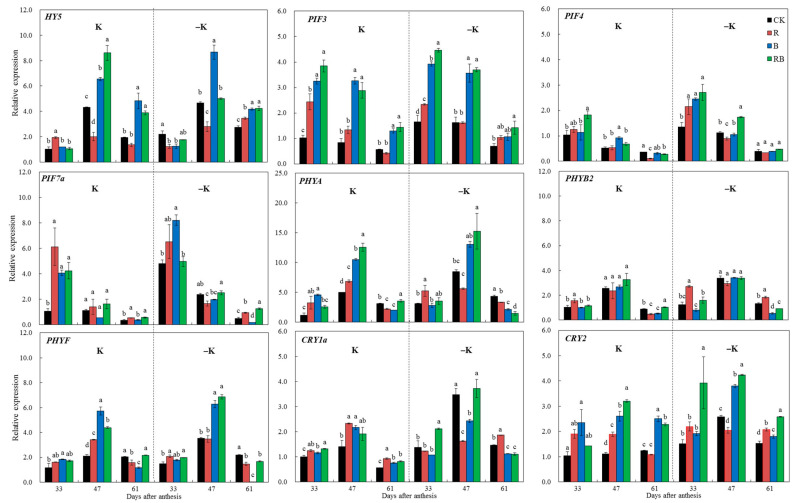
Expression of light interaction transcription factors and light receptors at 33, 42, and 61 DAA (days after anthesis) of tomatoes grown under red or/and blue supplemental lighting or without supplemental lighting at low K or normal K levels. The expression was normalized to the 33 DAA CK sample. Error bars represent standard deviations of the means of three independent replicates. Error bars represent standard deviations of the means of three independent replicates. Different letters indicate significant differences between treatments by Duncan’s multiple range test (*p* ≤ 0.05). CK: natural light, R: supplemental 660 nm red light, B: supplemental 430 nm blue light, RB: supplemental red combined with blue light with ratio of 3:1, K: normal potassium supply, –K: low potassium supply.

To explain how SL may induce an increased expression level of *SlHAKs*, we investigated light signaling transcription factors and light receptor genes. Most of the genes related to the light signaling pathway were up- or downregulated by SL, which was not related to the K supply level (Figure 6). PIFs and HY5 are light signaling components that mediate light responses in the tomato plant. As shown in Figure 6, *HY5* was continuously upregulated under blue SL from 47 to 61 DAA, but reduced by red SL at 47 DAA, which was not related to the K level. *PIF3* was increased by both blue and red combined blue SL from 33 to 47 DAA. *PHYA* was the most highly expressed light receptors, it was first increased by red at 33 DAA, then decreased at 61 DAA. *PHYA* was increased by blue and red combined blue at 47 DAA. *CRY1a* was increased by blue or red SL under normal K from 33 to 61 DAA, but repressed at DAA 47 by blue or red SL under low K supply. The expression levels of *PHYF* and *CRY2* were upregulated by blue or red combined blue SL at 47 DAA. 

### 2.7. Transcriptome Analysis 

**Figure 7:**
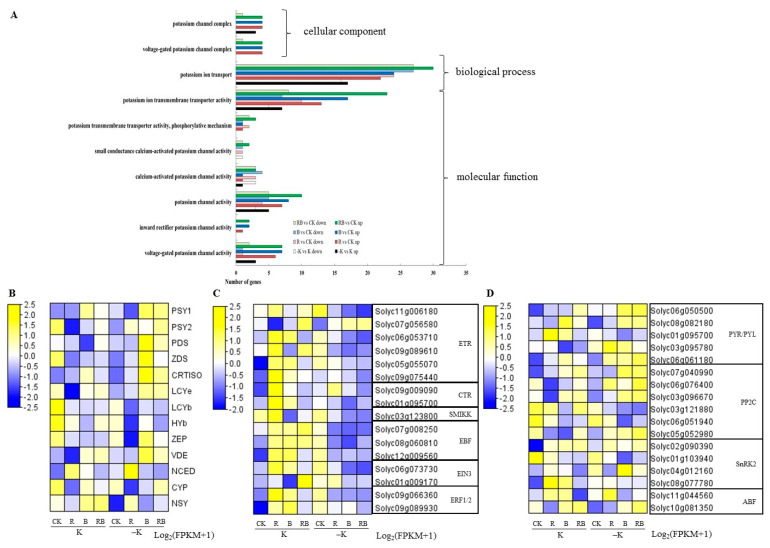
Transcriptome analysis of differentially expressed genes (DEGs) among supplemental lighting treatments or K levels in tomato fruits at 33 DAA (days after anthesis). (**A**) Up- and downregulated K channel and transporter related genes in supplemental red or/and blue light and low K treatments compared with CK; (**B**) DEGs assigned to fruit lycopene metabolism pathways between supplemental red or/and blue light treatment; (**C**) DEGs assigned to ethylene transduction pathways between supplemental red or/and blue light treatment; (**D**) DEGs assigned to abscisic acid (ABA) transduction pathways between supplemental red or/and blue light treatment. CK: natural light, R: supplemental 660 nm red light, B: supplemental 430 nm blue light, RB: supplemental red combined with blue light with ratio of 3:1, K: normal potassium supply, –K: low potassium supply.

From the transcriptome data of tomato fruit, we identified a total of 88 upregulated and 50 downregulated K transporter and channel differentially expressed genes (DEGs) between RB SL and CK, 68 upregulated and 46 downregulated between B SL and CK, 59 upregulated and 45 downregulated between R SL and CK (Figure 7A). Among them, K transporter and plasma membrane transporter activity were the largest, accounting for more than 60% of all the DEGs. The gene ontology (GO) enrichment analysis of all K transporters or channel genes showed that K transporter genes were mainly enriched in the classification of biological process and K channel genes were mainly enriched in molecular function (Figure 7A). 

The expression of the genes was normalized using fragments per kilobase of transcript per million mapped reads (FPKMs). The genes related to lycopene metabolism including *PSY1*, *PSY2*, *PDS*, *ZDS*, *CRITISO*, *LCYe*, *ZEP*, and *VDE* were all upregulated by blue or blue combined red SL at the −K level compared with CK (Figure 7B). *PSY1*, *CRTISO*, and *VDE* genes were upregulated by blue or blue combined red SL at normal K, which was much higher at low K supply condition compared with CK. *NSY* gene was upregulated by blue or blue combined red SL at normal K (Figure 7B). The GO enrichment analysis showed that the *NSY* gene was enriched in the K transport term (data not shown), suggesting that this gene may be involved in K transport, and its regulatory mechanism needs further study. The genes related to ethylene transduction (CTR) or receptors (ETR) were induced by red SL or low K treatment compared with CK (Figure 7C). The genes related to ABA signaling transduction were upregulated by blue or blue combined red SL at normal K, which was much higher at the low K supply condition compared with CK (Figure 7D). To verify the RNA-Seq data, qRT-PCR was conducted for the Sl*HAK*s. The qRT-PCR data were consistent with those obtained from the RNA-Seq experiment (Figure S4).

## 3. Discussion

### 3.1. Supplemental Lighting Increased K Uptake and Accumulation by Increasing Photosynthesis, Plant Growth, and Fruit Weight

The use of supplemental lighting increased the biomass and yield of tomatoes by increasing the fruit weight and enhancing plant growth [4]. In this study, the K accumulation, biomass, and yield of tomatoes were all increased by SL at both K supply levels (Figure 1A,C), and the increase percentage was higher in red SL or red combined blue SL than in blue SL. In comparison with red SL, reduction of plant growth and fruit yield of tomatoes under RB was associated with supplementation of blue light fraction. This was also reported in previous studies [27,28,29] where blue light promoted leaf peroxidation during the early-senescence phase and the product of membrane lipid peroxidation can ultimately contribute to leaf senescence [30]. 

In addition, high intensity blue light might suppress photosynthesis [31]. In this study, 100 μmol⋅m^−2^⋅s^−1^ of SL reduced the maximum photochemical quantum yield of PSII (Fv/Fm, Figure 2E), where blue SL was significantly lower than red SL, but the *Pn* of functional leaves was increased under SL treatments, and red SL was significantly higher than blue SL. These results indicate that SL induced slight photoinhibition, but PSII could still retain good use of the light captured. Valuations of the non-photochemical quenching (NPQ) indicated the impact of the light on the heat dissipation by leaves [32]. The present stronger decrease of NPQ under blue SL (Figure 2F) indicated a lower demand for energy dissipation, particularly after short illumination of dark-adapted tissues.

Consistent with enhanced plant growth, fruit yield, and photosynthesis, the K uptake rate of tomato roots (Figure 3B) was increased by SL, but K content in plant (Figure 1B) decreased. The K content in fruit increased when the rate of K accumulation was greater than fruit growth and development [33,34]. These results suggest that the rate of plant or fruit growth of tomatoes was much greater than the K accumulation rate in the present study.

K uptake by the cells of the root to shoot via the vessels was linearly related to water flux [35]. A recent study demonstrated that supplemental LED lighting increased tomato fruit growth through modulated root pressure [4]. In this study, the K uptake was higher under red SL during fruit development, but it was higher under blue SL than red SL during fruit ripening (Figure 3B). The higher uptake rate of K (Figure 3B) might be associated with increased *Pn*, *Gs*, and *Tr* under red SL light (Figure 2A,D). The use of supplemental lighting during the rapid fruit development stage of tomato was more efficient for the daily increase of yield than the fruit ripening stage [36]. Red light was also shown to increase K uptake in lettuce [37]. In previous reports, the adoption of blue light in broccoli and microgreens resulted in greater accumulation of K in leaf tissues compared with plants grown under mixed red and blue LED [38]. Altered fluxes of K in *Arabidopsis* were also associated with blue light [39]. 

Consequently, the use of red SL or red combined blue SL enhanced K uptake and accumulation by increasing photosynthesis, plant growth, and fruit weight.

### 3.2. Supplemental Lighting Induced Carotenoid and Accelerated Fruit Coloring of Tomato Fruits

K is the most enriched cation in tomato [10], and its requirement is extraordinarily high due to the rapid growth of the plant in combination with the heavy fruit load [12]. During tomato fruit ripening, the color changes from green to red or orange, which is a result of chlorophyll degradation as well as carotenoid synthesis, mainly including lycopene, phytoene, β-carotene, etc. [26]. Our previous research showed that K uptake rate was significantly correlated with fruit coloring [15]. 

In this study, there was a significant difference in the hue angle value (Figure 3C) among the different SL treatments at low K level, but it was not observed at normal K (Figure 3D). The uptake rate of K was correlated with the hue angle value at 61 DAA under both K levels, and at 47 DAA under low K level (Figure 3D). In addition, the contents of phytoene, γ-carotene, α-carotene, and β-carotene of tomatoes were significantly increased by blue SL or red combined blue SL under low K level, and lycopene content significantly increased under red combined blue SL, whereas this phenomenon was not observed at normal K supply (Figure 4). These results indicate that the blue or red combined blue SL treatment enhanced carotenoids and accelerated fruit coloring might be through enhancing K uptake at low K supply. 

In our previous research, red light increased lycopene content in tomatoes by inducing light receptors that modulate *HY5* and *PIFs* activation to mediate *PSY1* gene expression [8]. In the present study, phytoene, γ-carotene, and β-carotene of tomato fruits at 47 DAA was enhanced by red SL under both K supply levels (Figure 4). Correspondingly, the expression levels of *HY5*, *PIF3*, and *PIF7a* as well as photoreceptors *PHYB2*, *PHYA*, and *PHYF* in fruits were induced by red SL at 33 DAA (Figure 6), which was not related to K supply level. Ethylene regulates the production of phytoene synthase, which forms the first carotenoid, phytoene, from geranylgeranyl phosphate [40]. Red light results in the earlier ripening of tomato fruit depending on ethylene production [9]. In this study, the genes related to ethylene transduction (CTR) or receptors (ETR) were also upregulated by red SL compared with CK (Figure 7C). Consequently, enhanced phytoene and β-carotene by red SL might be mediated by ethylene signaling transduction in the present study. The genes related to lycopene metabolism including *PSY1*, *CRTISO*, and *VDE* were upregulated by blue and red combined blue SL at normal K, which was much higher at the low K supply condition compared with CK (Figure 7B). This was consistent with increased K average uptake rate at 61 DAA and enhanced level of phytoene, β-carotene, α-carotene and γ-carotene of tomato fruits under blue and red combined blue SL at low K supply (Figure 4B). These results indicated that, blue or red combined blue SL increased carotenoid content (such as phytoene, β-carotene, α-carotene and γ-carotene) in tomatoes by inducing photoreceptors that modulate HY5 and PIF3 activation to mediate carotenoid metabolism at low K supply. In addition, the key genes related to ABA signaling transduction were up-regulated by blue or red combined blue SL at normal K, which was much higher at low K supply condition compared with CK (Figure 7D). Carotenoids are precursors of ABA synthesis, and ABA can affect carotenoid metabolism and thus affect fruit color [41]. Therefore, enhanced phytoene and β-carotene under blue SL can also be explained by induced ABA signaling transduction at low K supply.

### 3.3. Potassium Transporter and Channel Genes in Tomato Fruits Were Upregulated by Blue Supplemental Lighting

The function of K in tomatoes depends on the effective transport and distribution of K^+^ in plants, among which K^+^ transporters or channels plays a key role [21,22,23,24]. The KT/KUP/HAK family comprises most of the plant K^+^ transporters identified thus far [42,43,44]. KT/HAK/KUP family genes are expressed throughout the plant including roots, leaves, stems, flowers, fruits, and seeds in plants [45,46,47], which are involved not only in primary K uptake from the soil, but also in cellular K homeostasis [48,49].

Nineteen KT/HAK/KUP family genes were identified in tomatoes, among which *SlHAK9* and *SlHAK16* were highly expressed in roots, and *SlHAK10* was highly expressed in leaves and fruits [25]. In this study, the expression level of *SlHAK10* was nearly 10-fold higher than the others when fruit reached breaker stage at 47 DAA, and maintained a high level until 61 DAA. *SlHAK3* and *SlHAK19* were highly expressed at 47 DAA, and *SlHAK18* was highly expressed at 61 DAA (Figure 5). K content in tomato fruits was the highest at 47 DAA, followed by 33 DAA, and the lowest at 61 DAA (Figure S2). Phylogenetic tree analysis showed that SlHAK10 was clustered with grape VvKUP2 and *Arabidopsis* AtKUP2, while SlHAK3 and SlHAK2 were clustered with peach PpKUP11 and *Arabidopsis* AtKUP10, SlHAK18, SlHAK19, and SlHAK1 were clustered together with PpKUP3 (Figure S3). *VvKUP2* was expressed most highly in the berry skin during the first phase of berry development, and the timing and location of its expression was consistent with an involvement in potassium accumulation in grape berries [21]. *PpKUP3* is continuously and stably expressed from young fruit stage to shelf life, and plays an important role in K nutrition and K homeostasis of peach fruit [23]. These results indicate that *SlHAK3*, *SlHAK10*, *SlHAK18,* and *SlHAK19* might be related to the formation of tomato fruit ripening and quality.

Gene members in Cluster I such as *SlHAK5*, *SlHAK12*-*14*, and *SlHAK16*-*17* were not detected in this study (Figure S2). Most identified *SlHAKs* were clustered into Clusters Ⅱ and Ⅲ. Transporters in this cluster have been characterized to mediate both high- and low-affinity K uptake [25]. In this study, *SlHAK2, SlHAK4, SlHAK6, SlHAK8, SlHAK9, SlHAK11, SlHAK15*, and *SlHAK18* were significantly induced by low K condition at 47 DAA (Figure S2), suggesting they have high-affinity K uptake capacity and rapid upregulation in response to K deficiency.

Under both K levels, the expression levels of *SlHAK6, SlHAK10*, *SlHAK15,* and *SlHAK19* were significantly higher in blue SL when fruits reached the breaker stage at 47 DAA compared with no SL (Figure 5). This is consistent with the average uptake rate of K and the hue angle value during the fruit ripening stage (Figure 3B,C). In contrast, increased K uptake rate in root under blue or red combined blue SL (Figure 3A,B) might be one reason for the result in upregulated *SlHAK6, SlHAK10*, *SlHAK15,* and *SlHAK19*. Blue light particularly stimulates proton/solute cotransport in bean leaves [50]. Blue light stimulates leaflet opening by inducing K^+^ release from the flexor motor cells [51]. The response to blue light includes a rapid depolarization of cell membrane potential [52]. The blue light could promote leaf peroxidation, and the product of membrane lipid peroxidation can ultimately contribute to leaf senescence [30]. In this study, another reason for induced *SlHAK6, SlHAK10*, *SlHAK15*, and *SlHAK19* by blue SL or red combined blue SL might be due to depolarization of the cell membrane, but further studies are needed to clarify it. 

In addition, the induction of photoreceptors, light signaling transcript factors as well as key genes in ABA transduction under blue and red combined blue SL were consistent with the upregulated genes of *SlHAK6*, *SlHAK10*, *SlHAK15,* and *SlHAK19* under blue and red combined blue SL. Previous research has reported that AtKUP6 subfamily transporters act as key factors in K homeostasis in both cell growth and drought stress responses mediated by ABA signaling [49]. The C-terminal of AtKUP6 is phosphorylated by the protein kinase SNF1-related protein kinase 2E of the ABA signaling receptor resistance family [49]. Consequently, the K transport in tomato fruits might be mediated by light signaling and ABA signaling transduction in the present study.

In addition to ABA, AP2/ERF (ethylene response factor) transcription factor RAP2.11 binds to the GCC-box site of the *AtHAK5* promoter, and its expression is affected by ROS, ethylene, and low K, and is involved in root growth and K uptake [53]. In this study, the key genes of ethylene transduction were upregulated by red SL compared with CK (Figure 7C). However, most of the K transporters or channels tested in the present study were not induced by red SL (Figure 5), which should be discussed in future research.

## 4. Materials and Methods

### 4.1. Plant Materials and Growth Conditions

Tomato (*Solanum. Lycopersicum* L. Micro-Tom) seedlings were cultivated on sponge blocks and fertigated with half strength Enshi formula nutrient solution, as described in our previous research [15]. The seedlings with three fully expanded true leaves were transplanted into a hydroponic system in an experimental greenhouse at South China Agriculture University (23.15868N, 113.34462E). Ten plants were kept in each container (40 cm × 55 cm × 11 cm) with 15 L Enshi formula nutrient solution, and renewed every seven days. Aeration was switched on for 15 min every hour. The average air temperature was 20 °C during greenhouse cultivation. 

### 4.2. Potassium and Light Treatments

The basal composition of the half strength Enshi formula nutrient solution was a control solution with normal K supply (Ca(NO_3_)_2_.4H_2_O: 475 mg⋅L^−1^, KNO_3_: 405 mg⋅L^−1^_,_ NH_4_H_2_PO_4_: 77 mg⋅L^−1^, MgSO_4_.7H_2_O: 247 mg⋅L^−1^, EDTA-2NaFe: 10 mg⋅L^−1^, H_3_BO_3_: 1.5 mg⋅L^−1^, MnSO_4_.4H_2_O: 1 mg⋅L^−1^, ZnSO_4_.7H_2_O: 0.11 mg⋅L^−1^, CuSO_4_.5H_2_O: 0.025 mg⋅L^−1^, and (NH_4_)_6_Mo_7_O_24_ ·4H_2_O: 0.01 mg⋅L^−1^). The KNO_3_ concentration was reduced to 101 mg·L^−1^ in the low K supply nutrient solution, in which a reduced amount of KNO_3_ was replaced by NaNO_3_. 

When tomato plants reached anthesis, the flowers were tagged every day and the plants were illuminated by the following SL light conditions: (1) CK: natural light, without any supplemental light; (2) R: supplemental red light (660 nm); (3) B: supplemental blue light (430 nm); and (4) RB: supplemental red and blue light (red: blue = 3:1). Photosynthetic photon flux density (PPFD) was set at 100 ± 5 µmol⋅m^−2^⋅s^−1^ and the illumination period extended from 06:00 to 18:00 h every day. A total of 24 hydroponics containers were used in this experiment. Ten plants were cultivated in each container. Every sixth container corresponded to one SL treatment. Each solution with three replicates was adopted, and each replicate contained 10 plants, thus 240 plants were used in this study.

### 4.3. Measurement and Statistical Analyses

#### 4.3.1. Growth Characteristics

Shoot length from the basal part to apical point and taproot length from the basal part to root tip were measured by a ruler 30 days after treatment. Stem diameter below the first true leaf and fruit diameter at 47 DAA (days after anthesis) were measured by a digital caliper. The chlorophyll content was measured by SPAD-502 Plus (Konica Minolta Business Associates Co. Ltd., Japan) 30 days after treatment. The fresh weight of shoots (including leaves and stem), roots, and fruits were recorded using an electronic balance 67 days after treatment, and the dry weight was recorded after plants were heated to de-enzyme at 105 °C, then dried at 80 °C until constant. The increase percentage of growth parameters was calculated SL and no SL improvement ratio of supplemental treatment to no supplemental light treatment at the same K level.

#### 4.3.2. K Content

Tomatoes were harvested at 33, 47, and 61 DAA, then dried in a forced air oven at 80 °C to constant weight, subsequently dry-ashed in a furnace at 550 °C for K content analysis. The content of total K was measured according to the method of flame photometry as described in our previous research [54].

#### 4.3.3. Photosynthetic Characteristics

The net photosynthetic rates (*Pn*), stomatal conductance (*Gs*), intercellular CO_2_ concentration (*Ci*), and transpiration rate (*Tr*) of the true leaf below first truss were measured by a Li-6400 photosynthetic system (L-COR, USA) at 10:00 on sunny days 30 days after treatment. A total of five fully expanded leaves from the five plants of each replicate were randomly selected for measurements.

#### 4.3.4. Maximum Quantum Efficiency of PSII and Non-photochemical Quenching 

Maximum quantum efficiency (Fv/Fm) of PSII and non-photochemical quenching (NPQ) of the whole plant (n = 3) were performed 30 DAT using a PlantExplorer^TM^ (PhenoVation B.V., Wageningen, Netherlands). The Fv/Fm indicates the maximum efficiency at which light absorbed by PSII is converted to chemical energy, but the NPQ provides an estimate of the heat dissipation by the leaves.

#### 4.3.5. Root K Uptake Rate 

In the greenhouse, the EC and pH of nutrient solution in each container was measured weekly (Figure S1). The level of K was investigated by an atomic absorption spectrometry method (AA900H, PerkinElmer Ltd., USA). K uptake rate per plant per week (mg⋅plant^−1^⋅week^−1^) was calculated as described in our previous research [15]. Average K uptake amount (mg⋅plant^−1^) during the fruit development and ripening period was calculated by average of the weekly amount from the first to fourth week and fifth to eighth week, respectively. 

#### 4.3.6. Fruit Coloring Measurement

Pericarp color of tomato fruits was assessed with a spectrophotometer (Konica Minolta CR-400, KONICA MINOLTA Ltd., Japan), as described in our previous research [8]. The hue angle value (in degrees, hue angle = tan^−1^(b*/a*), if a > 0; and 180 + tan^−1^ (b/a), if a < 0) was used to monitor the pericarp color of tomatoes, where the larger the value, the greener the fruit; conversely, the smaller the value, the redder the fruit. Tomatoes were harvested at 36, 47, and 61 DAA. For each time point, there were three biological replicates consisting of nine fruits. Each fruit was measured on three different points of its pericarp.

#### 4.3.7. Detection of Carotenoids

Fruit carotenoid composition and concentration were detected by MetWare (Wuhan, China). To measure carotenoid composition and concentration, the fruit pericarp of fresh tomato at 47 DAA were collected and stored at −80° until use. The samples were ground into powder. Fifty mg of sample was extracted with a mix of n-hexane:acetone:ethanol (2:1:1, *v*/*v*/*v*), and an internal standard was added. After two extractions, the supernatant was evaporated to dryness under a nitrogen gas stream, and reconstituted in a mixed solution of methanol:MTBE. The solution was filtered through a 0.22 μm filter for further liquid chromatography-atmospheric pressure chemical iosziaa-lion-tandem mass spectrometry (LC-APCI-MS/MS) analysis (UHPLC, ExionLC™ AD, https://sciex.com.cn/, accessed on 3 March 2021 [55]; MS, Applied Bio-systems 6500 Triple Quadrupole, https://sciex.com.cn/, accessed on 3 March 2021 [55]). A YMC C30 (3 µm, 100 mm × 2 mm) column was used for HPLC analysis. Samples were eluted using a gradient from solvent A, methanol:acetonitrile (3:1, *v*/*v*) added to 0.01% BHT and 0.1% formic acid), to solvent B, methyl tert-butyl ether (0.01% BHT). The analysis was carried out at 28 °C with a flow rate of 0.8 mL/min. MS analysis was performed using the API 6500 Q TRAP LC/MS/MS System, equipped with an APCI Turbo Ion-Spray interface, operating in a positive ion mode and controlled by Analyst 1.6.3 software. Carotenoid contents were detected by MetWare (http://www.metware.cn/, accessed on 3 March 2021 [55]) based on the AB Sciex QTRAP6500 LC-MS/MS platform.

#### 4.3.8. Total Ribosenucleic Acid (RNA) Isolation, Complementary Deoxyribonucleic Acid (cDNA) Synthesis, and Real-time PCR Analysis

Fruit samples collected at 36, 47, and 61 DAA were rapidly frozen in liquid nitrogen and stored at −80 °C until use. Each treatment sample comprised three biological replicates. Each replicate of nine fruits were collected from at least five different plants. Total RNA was isolated from samples by using the RNAprep Pure Plant Kit (Tiangen Biotech Co. Ltd., Beijing, China), and cDNA was synthesized from 1 μg of total RNA by using the PrimeScript TM RT Reagent Kit with gDNA Eraser (Perfect Real Time) (TaKaRa Bio, Inc., Shiga, Japan). qRT-PCR was performed using previously described methods [8]. A tomato polyubiquitin gene (UBQ) (Solyc01g056940) gene was used as the reference. The relative expression was normalized with the results of the mean values of Sl-UBI using the 2^−ΔΔCt^ method [56]. qRT-PCR was performed in three technical replicates for each sample. Primers used for qRT-PCR are listed in the Supplementary Materials in Table S1.

#### 4.3.9. RNA-Seq Analysis

Fruit samples at 33 DAA in each treatment were collected for RNA-Seq analysis. The pericarp was rapidly frozen in liquid nitrogen and stored at −80 °C until use. Five fruit from different plants in each treatment were pooled for each sample. Total RNA was extracted using the TRIzol method (Tiangen Biotech Co. Ltd., Beijing, China) and used for the production of an RNA-Seq library. Sequencing libraries were generated using the NEBNext^®^ Ultra™ RNA Library Prep Kit for Illumina^®^ (NEB, Ipswich, MA, USA) following the manufacturer’s recommendations and index codes were added to attribute sequences to each sample. Three biological replicates were performed for RNA transcriptome analyses. The library preparations were sequenced on an Illumina Hiseq 4000 platform by Beijing Allwegene Technology Company Limited (Beijing, China) and paired-end 150 bp reads were generated.

STAR software [57] was used to align the sequencing reads to the tomato genome (SL3.0). HTSeq v 0.5.4 p3 was used to count the read numbers mapped to each gene. Gene expression levels were estimated by fragments per kilobase of transcript per million fragments mapped (FPKMs). Differential expression analysis of two condition/groups was performed using the DESeq R package (1.10.1). Genes with an adjusted *p*-value < 0.05 found by DESeq were assigned as differentially expressed. GO enrichment analysis of the DEGs was implemented by the GOseq R package based Wallenius non-central hypergeometric distribution [58], which can adjust for gene length bias in DEGs.

### 4.4. Statistical Analysis

Significant differences among the treatments were determined by analysis of variance (ANOVA), followed by Duncan’s multiple range tests of SPSS 22.0 at *p* ≤ 0.05.

## 5. Conclusions

The use of red SL or red combined blue SL enhanced K uptake in roots and K accumulation as well as carotenoid content in fruits by increasing photosynthesis, plant growth, and fruit weight. The genes related to ethylene transduction or receptors were upregulated by red SL. Increased phytoene and β-carotene content by red SL might be mediated by ethylene signaling transduction. The expression level of *SlHAK6, SlHAK10*, *SlHAK15,* and *SlHAK19* were significantly increased by blue SL or red combined blue SL when fruits reached the breaker stage, which was not observed under red SL. Blue SL or red combined blue SL increased phytoene, β-carotene, α-carotene, and γ-carotene content and accelerated fruit coloring by enhancing K uptake in roots and transport in fruits during fruit ripening, which is consistent with the expression level of *SlHAK6*, *SlHAK10*, *SlHAK15*, and *SlHAK19* during fruit development and ripening. The key genes of photoreceptors, light signaling transcript factors as well as ABA transduction induced by blue SL or red combined blue SL were consistent with the upregulated genes of *SlHAK6*, *SlHAK10*, *SlHAK15,* and *SlHAK19* under blue and red combined blue SL. The K transport in tomato fruits might be mediated by light signaling and ABA signaling transduction.

## Data Availability

Data available in a publicly accessible repository.

## Supplementary Materials

The following are available online at https://www.mdpi.com/1422-0067/22/5/2687/s1, Figure S1: The EC (A) and pH (B) of nutrient solution in each container. Figure S2: Transcriptional analysis of the potassium transporter genes in tomato fruit at 33, 47, 61 DAA (days after anthesis) under normal and low potassium treatment. Error bars represent standard deviations of the means of three independent replicates. Different letters indicate significant differences between treatments by Duncan’s multiple range test (*p* ≤0.05). The asterisk * and ** indicate a significant difference at *p* ≤ 0.05, *p* ≤ 0.01, respectively. Figure S3: Phylogenetic tree of KT/HAK/KUP family proteins among tomatoes, Arabidopsis (At), rice (Os), grape (Vv) and peach (Pp). Figure S4: Confirmation of RNA-seq results by qRT-PCR. *SlHAKs* were analyzed. Table S1: Gene-specific primers used for quantitative real-time PCR analysis.
