# Supplementary material for: Effects of Supplemental Lighting on Potassium Transport and Fruit Coloring of Tomatoes Grown in Hydroponics
Source: Int J Mol Sci. 2021 Mar 7;22(5):2687. doi: 10.3390/ijms22052687 (PMC7961429; doi:10.3390/ijms22052687)
Supplement: Supplementary file 1 [file ijms-22-02687-s001.pdf]

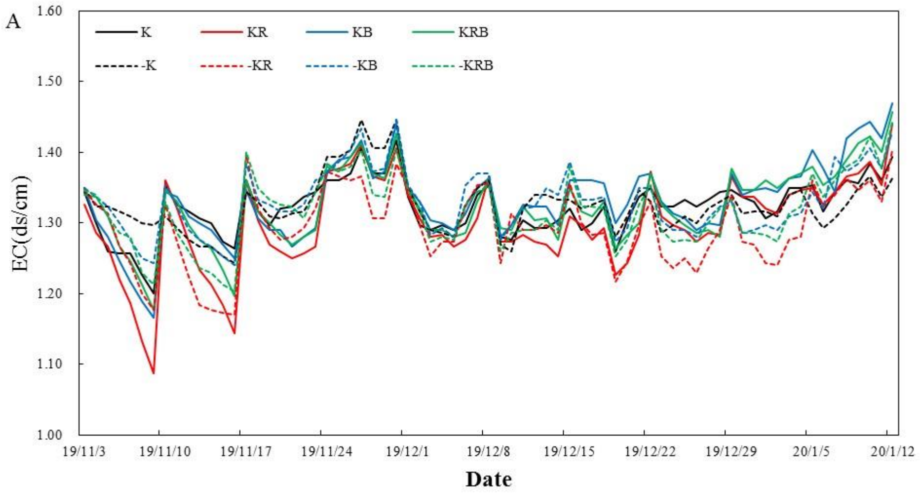

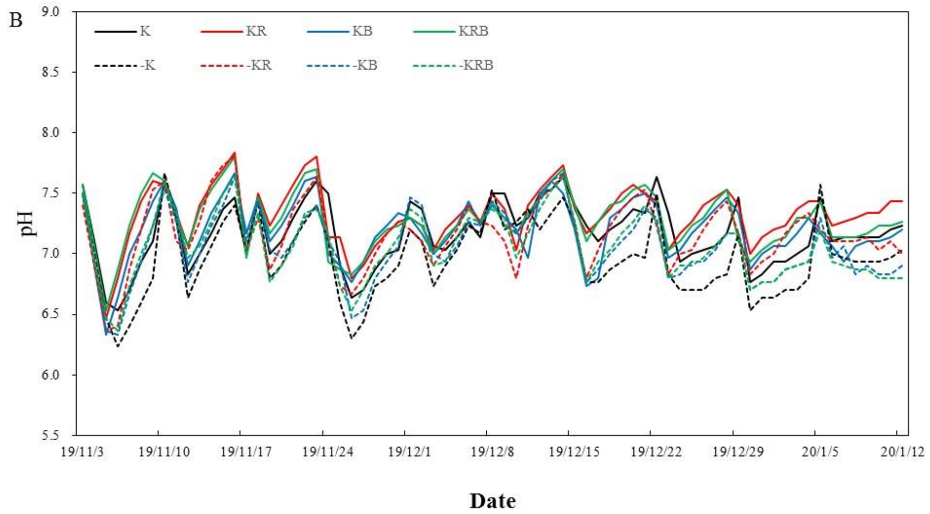

Figure S1. The EC (A) and pH (B) of nutrient solution in each container.

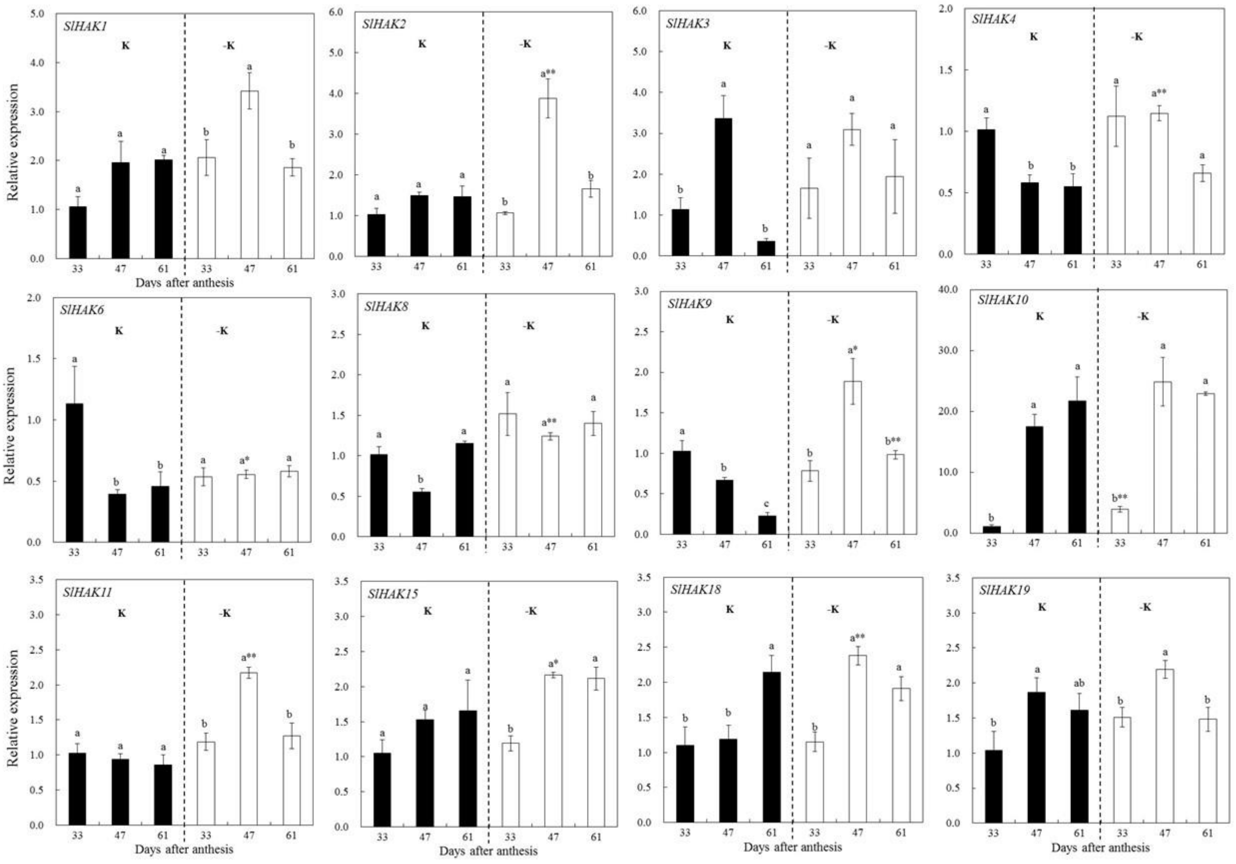

Figure S2. Transcriptional analysis of the potassium transporter genes in tomato fruit at 33, 47, 61 DAA (days after anthesis) under normal and low potassium treatment. Error bars represent standard deviations of the means of three independent replicates. Different letters indicate significant differences between treatments by Duncan's multiple range test (*p* ≤0.05). The asterisk ***** and ****** indicate a significant difference at p ≤ 0.05, p ≤ 0.01, respectively.

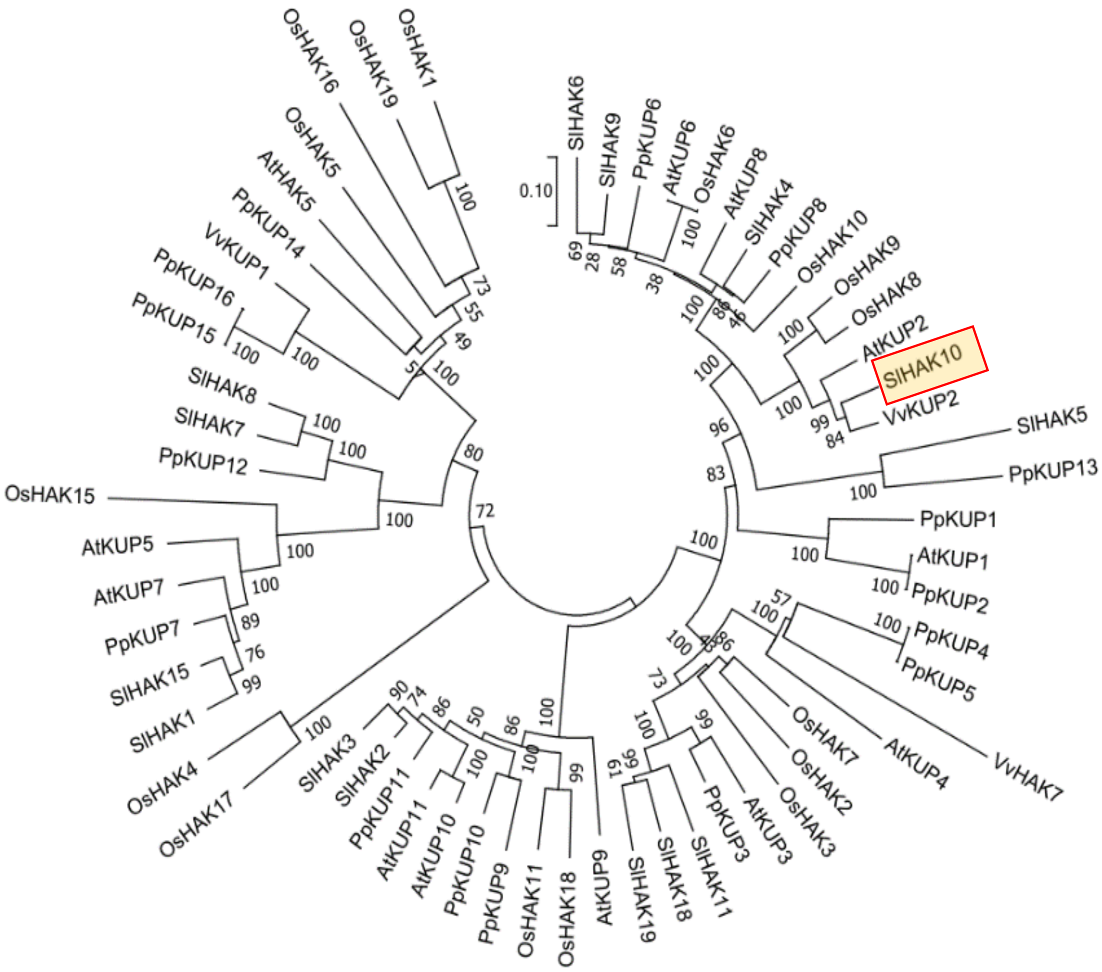

Figure S3. Phylogenetic tree of KT/HAK/KUP family proteins among tomatoes, *Arabidopsis(At)* , rice (Os), grape (Vv) and peach (Pp).

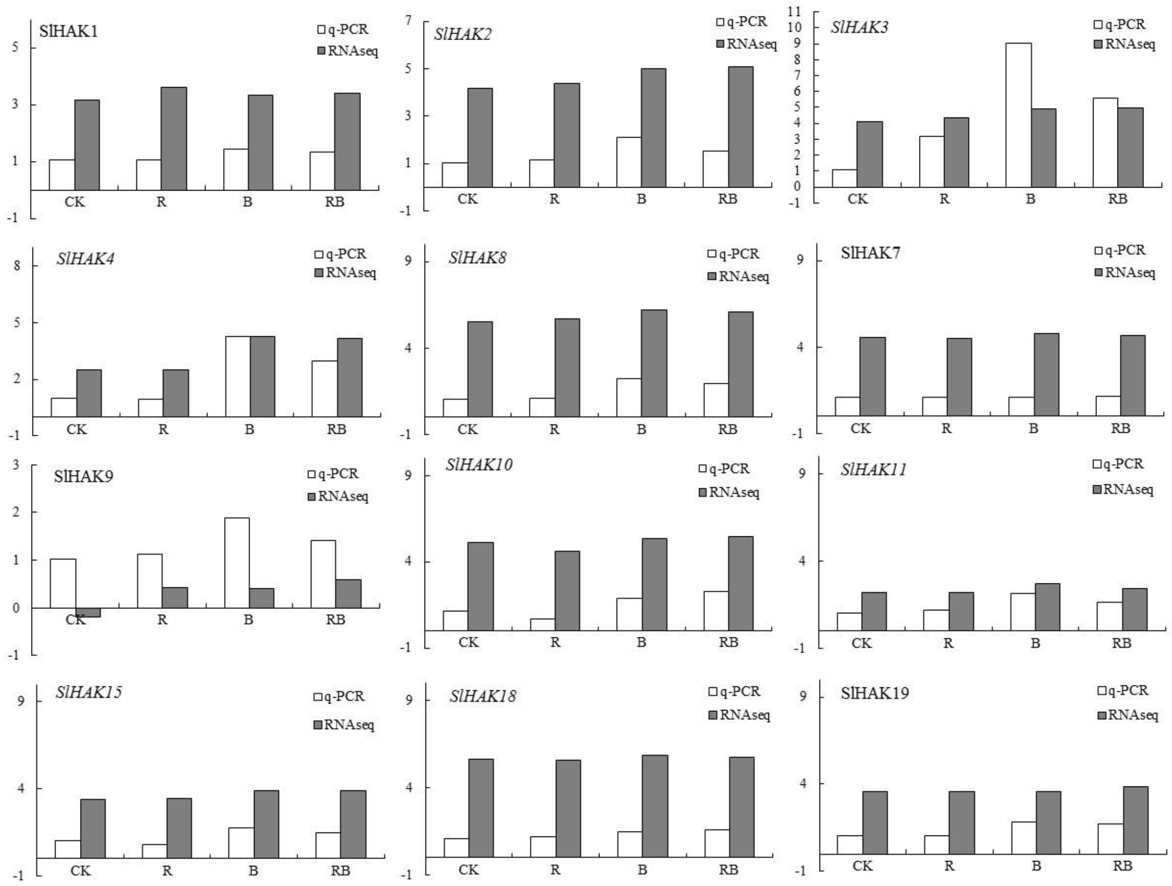

Figure S4. Confirmation of RNA-seq results by qRT-PCR. *SlHAKs* were analyzed.

Table S1.Gene-specific primers used for quantitative real-time PCR analysis.

Gene	Primer sequence 5'-3'
SlHY5	TCAACCATCAGCTGGGACTC (forward)
	CTCTCCCTTGCTTGTTGTGC (reverse)
SlPIF3	CCCGCCAATGATGTTCCCTA (forward)
	CAACCCAAATCCCATCCCCA (reverse)
SlPIF4	AGTGAAACAACGGCCACAGA (forward)
	GGGAAAACAAACCAAGTCAGGG (reverse)
SlPIF7a	CATGGCAGCTTTGTATCGGC (forward)
	GGTTCAACTTGCTGCCTGTG (reverse)
SlPHYA	TGCTAATTTTGGGATGACACCA (forward)
	GACGACATCTCGACTCAAATCTAAA (reverse)
SlPHYB2	TCCTTATGCAGGCCTTTGGG (forward)
	TTTTCAGCCAACTGCGATGC (reverse)
SlPHYF	TGATGCTTGTTCACATGGAGGA (forward)
	ACAGCAAGAATCCACCAAATTCA (reverse)
SlCRY1a	CAGCGGCTTTCTCAAACAGG (forward)
	TCTCCGCCTTGTTGCTATCG (reverse)
SlCRY2	TTGCCCGAGCAGTTCATCTT (forward)
	TTCCTCTTCGGGCTTTGGTC (reverse)
SlKAT1	GCAGAATGAGGCACCAACTG(forward)
	TCCTGCAACGGCTTTTCCAA(reverse)
SlAKT2/3	CCGATCCCTACATTGCTGGT(forward)
	TTGATCCATCGCGGTCCTTC(reverse)
SlKLT1	GCAAATCCAGAAGATGAACGGAT(forward)
	GGTCCTGCGTTTGATTGGTG(reverse)
SlHAK1	CGCATTTAGTGCAGATATTGTCAT (forward)
	CCAGAAGCCAAATCTTCCAAGC (reverse)
SlHAK2	TGTTGGCACCTGCATGGTAA (forward)
	GTCCACCTTGATCCCACCAG (reverse)
SlHAK3	AGGAGGAATTGCAGAGGTGC (forward)
	TTTGAAGCTACACGCCATGC (reverse)
SlHAK4	GAAGCCAGGCCATCATCACT (forward)
	CTTGGGAAGCAACCCAAAGC (reverse)
SlHAK6	GTTGGAACGTCGAAGCACAC (forward)
	TCGCACAACTCCTCTCTTGC (reverse)
SlHAK8	TGTCTGCTGTTAGCGGACTG (forward)
	AACAACCCGCCAAGGATGAT (reverse)
SlHAK9	TAACGTGGGACCGTCATGTG (forward)
	AGGACATCCAGCCTCCTCTT (reverse)
SlHAK10	CTGGTGGGATCAAGGTGGAC (forward)
	AACCAACCCTGTCTGTTCCG (reverse)
SlHAK11	ATATGGCTTTGCAGGGCAGT (forward)
	ATAACCATGCAAGCCCCCAA (reverse)
SlHAK15	CGTGAACTAGGCCCAAACCT (forward)
	TGGTATGCCCTTTGCCAGTT (reverse)
SlHAK18	CGGTCGCCGACGAATAGATG (forward)
	CAGGAGAAGGTGGTTGCTCC (reverse)
SlHAK19	AGCTTCCGCTGTTGCACTTA (forward)
	CTTGGCTCCAAGCCTCTCTC (reverse)
SlUBI	CCAAGATCCAGGACAAGGAA (forward)
	AAATCAAACGCTGCTGGTCT (reverse)
